# An investigation of whether patients with post-traumatic stress disorder overestimate the probability and cost of future negative events

**DOI:** 10.1016/j.janxdis.2008.01.004

**Published:** 2008-10

**Authors:** Melanie White, Freda McManus, Anke Ehlers

**Affiliations:** a Lowestoft Community Mental Health Team, Norfolk & Waverley NHS Foundation Trust, Victoria House, 28 Alexandra Road, Lowestoft, NR32 1PL, United Kingdom; b Oxford Cognitive Therapy Centre and University of Oxford Department of Psychiatry, Warneford Hospital, Oxford, OX3 7JX, United Kingdom; c Psychology Department, Institute of Psychiatry, King's College London, United Kingdom

**Keywords:** Probability, Cost, Anxiety disorders, Post-traumatic stress disorder

## Abstract

This study compared estimations of the probability and cost of negative events occurring made by patients with post-traumatic stress disorder (PTSD) (*n* = 43), patients with other anxiety disorders (*n* = 29) and non-patients’ (*n* = 35). Prior to treatment PTSD patients overestimated the probability and cost of all types of traumatic events occurring relative to non-patients, and overestimated the probability and cost of the specific type of traumatic event that they had been traumatized by relative to the anxious controls as well as non-patients. These judgment biases were specific to traumatic events and did not generalise to all negative events. PTSD patients’ estimations of the probability and cost of traumatic events were significantly reduced following treatment, and were no longer significantly different from those of non-patients. Results suggest that patients with PTSD show specific judgment biases in the estimation of probability and cost, which can be successfully modified by cognitive therapy.

## 1 Introduction

Post-traumatic stress disorder (PTSD) is a common and disabling anxiety disorder (Yehuda, 2001). In recent years several cognitive models of PTSD have been proposed (see Brewin & Holmes, 2003, for a review). Some of these cognitive models of PTSD are based on the generic cognitive model of anxiety (Beck, 1976) that posits that anxiety results from the appraisal of future threat. However, in the case of PTSD the trauma has already happened and thus the threat is no longer impending. Ehlers and Clark's cognitive model of PTSD (2000) attempts to account for this anomaly by proposing that individuals who experience persistent PTSD interpret the trauma or its sequelae in maladaptive ways that give rise to a sense of current threat. These idiosyncratic interpretations may relate to the external world (e.g., “I will be assaulted again”), to themselves (e.g., “this event/my reactions to it show that I am inferior to others”), to the future (e.g., “I will never be able to lead a normal life or be happy again”) or to the meaning of their symptoms (e.g., “these symptoms show what a weak person I am”).

### 1.1 Estimations of probability and cost

Beck (1976) proposed that perceived danger or threat is substantially determined by the joint product of the subjective probability and cost of a feared event. Studies have shown that increased estimates of the probability and cost of certain types of negative events are present in generalized anxiety disorder (Butler & Mathews, 1983), social phobia (Foa, Franklin, Perry, & Herbert, 1996; McManus, Clark, & Hackmann, 2000), and agoraphobia (McNally & Roa, 1987). Some of these studies have reported content-specificity of these judgment biases. For example, people with social phobia overestimated the probability and cost of future negative social events occurring, but not negative non-social events (Foa et al., 1996). However, Smith and Bryant (2000) reported that traumatized adults with acute stress disorder (ASD) overestimated the probability and cost not only of dangerous external events, but also of negative social events and negative somatic events. They concluded that because the experience of trauma leads to the development of wide reaching and dysfunctional fear networks, the traumatized individual's sense of threat extends far beyond the context of the original trauma.

The only study that has looked at estimations of probability and cost in PTSD was carried out with children. It found that children with PTSD did not overestimate the probability of future negative events occurring, such as being bullied at school, in comparison to controls (Dalgleish et al., 2000). Although this is in contrast to Smith and Bryant's (2000) report of overestimations of probability and cost in ASD, it is consistent with other studies where children failed to show the biases in probability and cost reported in adult samples (Dalgleish et al., 1997).

A further line of research has investigated effects of treatment on biases in estimations of probability and cost. This research suggests that such biases in social phobia can be attenuated by cognitive therapy (Lucock & Salkovskis, 1988) though not necessarily to the level shown by non-patients (e.g., McManus et al., 2000). It is not clear whether reductions in the overestimation of probability or cost are most closely related to improvement in treatment, with some studies reporting reductions in cost of negative events being most closely associated with treatment outcome (e.g., Foa et al., 1996) and others finding that reductions in the overestimation of probability are more closely linked to improvement in treatment (McManus et al., 2000). It is not know whether treatment outcome in PTSD is associated with corresponding changes in the estimations of probability or cost of negative events.

The current study has two key aims: first, to investigate whether adults with PTSD overestimate the probability and cost of being involved in future traumatic and/or negative non-traumatic events, relative to patients with other anxiety disorders and non-patients, and second to examine the extent to which these judgment biases are modified by cognitive therapy and whether changes in estimation of probability or cost are associated with improvement in treatment.

## 2 Method

### 2.1 Participants

Three groups of participants took part in the study: patients with PTSD, an anxious control group comprising patients suffering anxiety disorders other than PTSD, and non-patient controls. All participants were aged between 18 and 65 years. Patients were recruited from the Centre for Anxiety Disorders and Trauma, Maudsley Hospital, London, UK, an outpatient specialist National Health Service clinic that receives referrals from family doctors and community mental health clinics. Inclusion criteria for the clinic are that the patient's main problem is an anxiety disorder. Patients were diagnosed with the Structured Clinical Interview for DSM-IV (First et al., 1995) and met DSM-IV diagnostic criteria. Exclusion criteria for the present study were that the patient suffered from one of the following disorders to such a degree that it required immediate treatment in its own right: psychosis or bipolar disorder, alcohol or drug dependence, severe depressive disorder (i.e., immediate suicide risk) or borderline personality disorder. For the PTSD group, additional exclusion criteria were (i) severe ongoing threat (i.e., that the person continued to be in unacceptable level of personal danger as a consequence of the trauma), and (ii) an inability to remember the traumatic event. Patients with multiple traumatic events and those that had failed to respond to previous treatments were included. The types of traumatic event experienced by the PTSD group included serious traffic accidents, torture, sexual and non-sexual assaults.

The PTSD group consisted of 43 participants (15 men) about to start a course of cognitive therapy for PTSD (Ehlers et al., 2003; Ehlers, Clark, Hackmann, McManus, & Fennell, 2005). Of the 43 participants who took part in the pre-treatment phase, 36 agreed to repeat the questionnaires post-treatment. Of the seven participants who did not repeat the questionnaires, two had discontinued treatment pre-maturely, and five participants declined participation in the post-treatment phase.

The anxious control group consisted of 29 participants (7 men) about to start cognitive therapy for an anxiety disorder other than PTSD. Twenty had a primary diagnosis of panic disorder, five had a primary diagnosis of generalized anxiety disorder, two had a primary diagnosis of obsessive compulsive disorder, and two had a primary diagnosis of social phobia. All participants were screened to rule out a diagnosis of co-morbid PTSD.

The non-patient control group consisted of 35 participants (15 men), not currently receiving treatment for a psychiatric disorder. These participants were recruited from the local community. Participants in the non-patient group were screened for experiences of trauma and PTSD using a trauma checklist, followed by the Posttraumatic Diagnostic Scale (PDS; Foa, Cashman, Jaycox, & Perry, 1997) and would have been excluded from the study if they met full diagnostic criteria for PTSD.

### 2.2 Design

This was a questionnaire study with a two-fold design. The first step involved a between-subjects comparison of the three groups (pre-treatment PTSD patients vs. other anxiety disorder patients vs. non-patients). The second step, a repeated measures design, involved comparing the PTSD group's estimates of probability and cost before and after receiving treatment, and examining the relationship between changes in these estimates with symptomatic improvement following treatment.

### 2.3 Measures

#### 2.3.1 Demographic information

Collected on all participants including: age, level of educational achievement, race, and gender.

#### 2.3.2 Symptom measures

Patients and controls who reported a traumatic event completed the Posttraumatic Diagnostic Scale (PDS; Foa et al., 1997), which asks patients to rate how much they experienced each of the PTSD symptoms identified in DSM-IV, ranging from 0 (“never”) to 3 (“5 times per week or more or almost always”). The PDS yields a sum score indicating the overall severity of PTSD symptoms Foa et al. (1997) demonstrated that the PDS has good reliability and concurrent validity with other PTSD measures and with the structured clinical interview for DSM-III-R (Foa et al., 1997). To check the comparability of the three groups all participants were asked to complete a standardized measure of anxiety, the Beck Anxiety Inventory (BAI; Beck & Steer, 1993) and of depression, the Beck Depression Inventory (BDI; Beck, Steer, and Brown, 1996). Patients with PTSD also completed the symptom measures again after treatment.

#### 2.3.3 Probability and Cost Questionnaire (PCQ)

This questionnaire (McManus & Ehlers, unpublished measure—see Appendix A) was developed for the purposes of this study. The PCQ consists of five subscales of four items, each of which describes a potential future negative event. Participants are asked to rate how likely each event is to happen to them in the near future on a scale from 0 ‘not at all likely to happen’ to 100 ‘almost sure to happen,’ and how bad or distressing it would be if it did happen on a scale from 0 ‘not at all bad/distressing’ to 100 ‘really bad/distressing.’ The five subscales were as follows: accidental traumas (e.g., ‘I will be involved in a road traffic accident’), interpersonal violence traumas (e.g., ‘I will be mugged’), negative life events (e.g., ‘a friend of mine will die’), negative world events (e.g., ‘there will be a famine that affects a third world country’) and daily hassles (e.g., ‘I will get caught out in the rain’). The internal consistency of each of the subscales was relatively high with Cronbach's alpha coefficients ranging from 0.71 to 0.92 for both probability and cost ratings on each of the five subscales.

In addition to the five sub-scales described above, a further two subscales were derived for the PTSD group according to the type of event that they had been traumatized by. These two scales were “own type trauma” (i.e., for those who were traumatized by an accident this would be their score on the accidental traumas subscale), and “other type trauma,” (i.e., for those who were traumatized by an accident this would be their score on the interpersonal violence trauma sub-scale).

### 2.4 Data analysis

Each hypothesis was analysed in turn. Kolmogorov–Smirnov Tests were used to examine whether the data was normally distributed and Levene's test for equity of variance was used to look at the spread of the data. Where the data violated the assumptions for the use of parametric tests, transformations were attempted to alter the distribution. Where no suitable transformation could be found non-parametric equivalents were used.

The between group analyses were conducted using either a one-way analysis of variance (ANOVA) with Scheffe post hoc analyses, or a Kruskal–Wallis test, with Mann–Whitney tests used for post hoc analyses. Paired sample *t*-tests were used to conduct the within-group analyses and the repeated measures design. Pearson's correlations and partial correlations were used to examine the relationship between improvement in treatment and changes in probability and cost estimates.

## 3 Results

### 3.1 Demographic information

Participant demographics are shown in Table 1:

There were no significant differences between the groups in terms of age, gender, ethnicity or marital status. There was a significant difference between the groups in terms of educational level. There was a greater proportion of graduates in the non-patient and anxious control groups, compared to the PTSD pre-treatment group. Thus where differences between the PTSD group and the other groups were found Analyses of Covariance (ANCOVA) were used to check that the differences held up when education was controlled. Within the PTSD group, the graduates did not differ from the non-graduates on PDS scores (*t* = 1.3, *p* = ns) and or on ratings of probability (*t* = 0.11, *p* = ns) or cost (*t* = −0.3, *p* = ns).

Table 2 shows the group means and standard deviations on the BDI, BAI, and PDS.

As expected, pre-treatment clinical groups scored significantly higher than the non-patient group on all of the clinical measures: BDI (*H* = 56.75, *p* < 0.001), BAI (*H* = 70.59, *p* > 0.001) and PDS (*H* = 72.82, *p* > 0.001). No significant differences were found between the PTSD pre-treatment group and the anxious control group on the BDI or the BAI, suggesting that the two groups showed similar levels of clinical severity.

### 3.2 Probability and cost of experiencing a traumatic event

Group means and standard deviations for the probability and cost of experiencing a traumatic event or other negative event are shown in Table 3.

A significant difference in estimations of probability (*F*(2,96) = 3.19, *p* = 0.046) and cost (*F*(2,93) = 4.65, *p* = 0.013) was found between the groups. Post hoc tests indicated that PTSD patients overestimated the probability and cost of experiencing traumatic events relative to the non-patient controls, but not relative to the anxious controls. This effect remained when the effect of education was controlled (for probability *F*(2,96) = 4.01, *p* = 0.021; for cost *F*(2,93) = 3.63, *p* = 0.03) and the effect of education was not significant. The anxious control group did not differ significantly in their estimations from either the PTSD group or the non-patient group.

### 3.3 Own type trauma versus other type trauma

The PTSD group's mean scores for the probability (mean = 37.15; S.D. = 27.11) and cost (mean = 83.63; S.D. = 19.31) of experiencing another trauma of the same type as their previous trauma (‘own type of trauma’) were compared with their mean scores for the probability (mean = 29.84; S.D. = 22.54) and cost (70.29; 26.08) of experiencing a trauma of different type (‘other type of trauma’). The PTSD group rated both the probability (*t* = 2.60, *p* < 0.05) and cost (*t* = 5.27, *p* < 0.05) of experiencing their own type of trauma again higher than the probability and cost of experiencing a different type of trauma.

The PTSD group's ratings of the probability (mean = 37.15; S.D. = 27.11) and cost (mean = 83.63; S.D. = 19.31) of experiencing their own type of trauma was then compared with the anxious control group's ratings of the probability (mean = 24.05; S.D. = 19.73) and cost (mean = 77.83; S.D. = 18.52) of experiencing any type of trauma. Independent *t*-tests showed that the PTSD group gave significantly higher rating for their own type of trauma occurring than the anxious control group gave for any type of traumatic event occurring (*t* = −2.20, *p* < 0.05). Although the difference in cost ratings was in the expected direction, it was not significant (*t* = −1.170, *p* = ns).

### 3.4 Probability and cost of experiencing a negative, non-traumatic event

Group means and standard deviations for the probability and cost of experiencing a negative, non-traumatic event are given in Table 3. No significant differences were found between the three groups on their estimations of the probability of future negative life events (*U* = 4.39, *p* = ns), negative world events (*F*(2,104) = 1, *p* = ns), or daily hassles (*F*(2,104) = 0.45, *p* = ns) occurring.

No significant differences were found between the three groups on their estimates of the cost of future negative life events (*F*(2,99) = 3.04, *p* = ns) or negative world events occurring (*F*(2,98) = 1.84, *p* = ns). Even when education was controlled there was a significant effect of group on the rating of the cost of daily hassles (*F*(2,97) = 4.63, *p* = 0.012) and the effect education was not significant. Post hoc tests revealed that PTSD patients rated the cost of daily hassles significantly higher than either of the control groups (*U* = 7.51, *p* < 0.05).

### 3.5 Effect of treatment on symptoms and estimations of probability and cost

The means and standard deviations of the PTSD group's questionnaire scores pre and post-treatment are given in Table 4.

PTSD patients scored significantly lower on the PDS, BDI, and BAI following treatment. Dependent *t*-tests showed that the PTSD group scored significantly lower on all five sub-scales of the Probability and Cost Questionnaire at post-treatment than pre-treatment. To see if the PTSD group's estimations of the probability and cost of traumatic events occurring had returned to non-clinical levels their post-treatment scores on the trauma subscales of the Probability and Cost Questionnaire were compared with those of the non-patient control group. Independent sample *t*-tests showed that there were no significant differences between the post-treatment PTSD group's scores and the non-patients’ scores for the probability of experiencing an accidental trauma (*t* = 0.26, *p* = ns) or an inter-personal violence trauma (*t* = −0.48, *p* = ns) or for the cost of experiencing an accidental trauma (*t* = −0.76, *p* = ns) or an inter-personal violence trauma (*t* = 0.45, *p* = ns).

### 3.6 The relationship between changes in estimations of probability and cost and treatment outcome

The PDS was repeated after treatment to assess the impact of treatment on patients’ PTSD symptoms. PTSD patients’ scores on the PDS were significantly reduced by treatment (*t* = 8.86, *p* < 0.001). A Pearson's correlation was used to see how changes in estimations of the probability and cost of being involved in future traumatic events were related to change in PDS scores post-treatment. There were significant correlations between change in PDS scores and change in both probability and cost estimations (*r* = 0.398, *p* = 0.024 and *r* = 0.439, *p* = 0.011 respectively). The correlation between change in probability estimates and change in PDS scores did not remain significant when change in cost estimates was controlled for (*r* = 0.146, *p* > 0.05). The correlation between change in cost estimates and change in PDS remained significant when changes in probability estimates were controlled for (*r* = 0.401, *p* = 0.038).

## 4 Discussion

Results of the current study show that, prior to receiving treatment, patients with PTSD overestimate the probability and cost of being involved in future traumatic events relative to non-patient controls. They particularly overestimate the probability and cost of traumatic events of the same type as the event that triggered their PTSD. This overestimation of the probability and cost of ‘own’ traumas occurs in comparison to non-anxious controls, in comparison to the anxious control group, and also in comparison to their own estimations of the probability and cost of ‘other’ traumatic events. This evidence of overestimation of the probability and cost of future traumatic events is in line with cognitive models of PTSD (e.g., Ehlers & Clark, 2000). The finding that PTSD patients overestimate both the likelihood and cost of future traumatic events occurring suggests that they are interpreting the fact that the trauma occurred as indicative of a current or future threat. It also seems that there is some specificity to the source of this threat, as PTSD patients did not show overestimations of the probability and cost of all types of negative event.

Previous research has suggested that patients with different anxiety disorders show unique, disorder specific patterns of judgment bias (McManus et al., 2000), which are not attributable to general anxiety. In this study, the PTSD group indeed showed such a specific bias in that they overestimated the probability and cost of further traumas of the type that they had experienced. However, contrary to predictions, the PTSD group did not overestimate the probability and cost of all traumatic events relative to patients with other anxiety disorders, although means for probability ratings were in the expected direction. Low power may have been in part responsible for the lack of significance for traumatic events in general. Another contributing factor may be that 20 of the 29 participants in the anxious control group had a primary diagnosis of panic disorder; and it has recently been suggested that patients with panic disorder showed more generalized biases in overestimation of negative events that other anxiety disorder patients (Uren, Szabo, & Lovibond, 2004). Nevertheless, patients with PTSD estimated the cost and probability of the type of traumatic event that matched their own trauma compared to an equally anxious and depressed control group, ruling out the possibility that this judgment bias merely reflects elevated anxiety or depression.

In contrast to Smith and Bryant's (2000) finding of generalized biases in ASD patients, the PTSD patients in this study only showed a specific bias for the types of traumatic event that they had experienced. They did not overestimate probability or cost of all negative non-traumatic events. These findings are in keeping with studies of social phobia (Foa et al., 1996) and eating disorders (Cooper, 1997), where very specific judgment biases have been reported. A possible explanation of the finding of highly generalized biases in ASD but very specific biases PTSD is that ASD is a reaction that occurs in the first four weeks following the trauma. At this time the person is still in a heightened state of distress and processes of adjustment are in their early stages. In this initial phase the cognitive biases shown may be generalized and wide reaching; however, as time progresses and some adjustment takes place, the cognitive biases become more specific and fixed on stimuli specific to the trauma. This is a preliminary hypothesis that requires further investigation with longitudinal studies.

It is important to note that prior to treatment, the PTSD pre-treatment group overestimated the cost of daily hassles relative to both the anxious control group and the non-patient group. Overestimation of the cost of daily hassles may reflect difficulties that people experiencing chronic PTSD are likely to be having functioning in their day-to-day life, in that they may find daily hassles more difficult to cope with than others because of other consequences of the trauma/PTSD impacting on their life (e.g., financial problems, physical disabilities or pain).

Prior to treatment, PTSD patients overestimated the probability and cost of all types of traumatic events occurring relative to non-patients. After treatment PTSD patients showed reduced estimations of probability and cost on all of the sub-scales, and their estimations were no longer significantly different from the non-patient group on any subscale of the Probability and Cost Questionnaire. This is a positive result, which adds to the existing evidence suggesting that cognitive therapy for PTSD is effective at addressing the cognitive biases that underlie PTSD (Ehlers et al., 2005). Change in both probability and cost estimations were found to be a significantly correlated with changes in PTSD symptoms (as measured by the PDS), suggesting a link between reductions in cognitive biases and a reduction in symptoms. However, only the correlation with changes in cost estimations remained significant when controlling for probability estimates. This suggests that it may be more important for clinicians to focus on modifying PTSD patients’ appraisals of the cost of future traumatic events in order to achieve good outcomes from CBT treatment.

Current results need to be interpreted in the light of some of the limitations of the study. One limitation of the study is that this is the first time that the Probability and Cost Questionnaire has been used and hence, there is only limited information available about its validity and reliability. However, results suggest that the questionnaire shows good internal consistency and clinical sensitivity, i.e., it was able to distinguish clinical groups from non-clinical groups. The questionnaire also appeared to have reasonable construct validity, as evidenced by participants rating daily hassles as more likely to happen than inter-personal violence traumas and negative life events as more personally costly than negative world events. Larger samples, particularly in the post-treatment phase, would also have strengthened the study by giving more power for some of the analyses. Another limitation of the study is that the groups were not matched for educational level (a smaller proportion of the PTSD patients were educated to graduate level than in the other two groups). This finding matches the literature showing that lower intelligence/education predicts PTSD (McNally & Shin, 1995). And it is worth noting that there were no differenced in ratings of probability and cost between the more and less educated PTSD patients, and any difference between the PTSD patients and the control groups remained when level of education was controlled for statistically.

Findings of the current study support both the cognitive model of PTSD and the role of cognitive therapy in the treatment of PTSD. The finding of a specific overestimation of the probability and cost of the type of trauma that the patient was traumatized is consistent with the central premise of Ehlers and Clark's (2000) cognitive model of PTSD, i.e., that the trauma is interpreted as a current or future threat. Current results also support the validity of cognitive therapy for PTSD as an effective way of modifying PTSD patients’ overestimations of the probability and cost of future traumatic events.

## Figures and Tables

**Figure:**
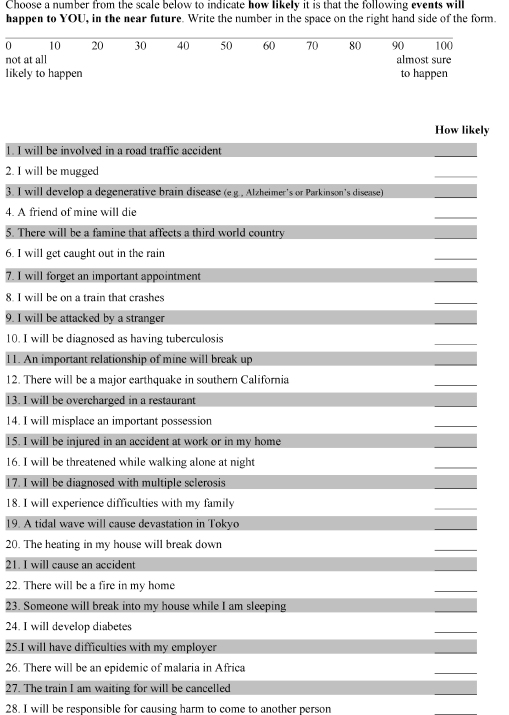


**Figure:**
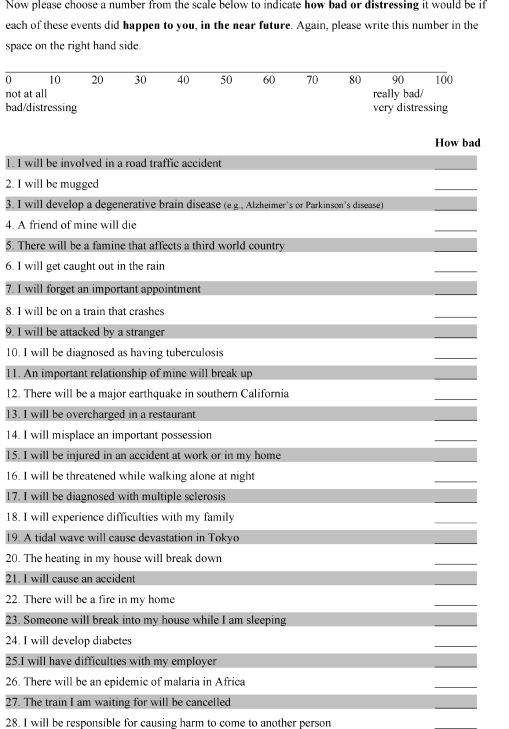


**Table 1:** Participant demographics

Demographic variable	PTSD pre-treatment group (*N* = 43)	Anxious control group (*N* = 29)	Non-patient control group (*N* = 35)
Age			
Mean age in years (S.D.)	36.9 (11.4)	37.1 (11.7)	40.7 (13.5)

Gender			
No. of males (%)	15 (34.9)	7 (24.1)	13 (37.1)
No. of females (%)	28 (65.1)	22 (73.9)	22 (62.9)

Ethnic origin			
No. (%)			
Caucasian	32 (74.4)	23 (79.3)	27 (77.1)
Non-Caucasian	11 (25.6)	6 (20.7)	8 (22.9)

Level of education			
No. of achieving (%)			
GCSE/O’level	21 (50.0)	7 (24.1)	7 (20.0)
A’level/BTEC	11 (26.2)	3 (10.3)	5 (14.3)
Degree/HND	10 (23.8)	19 (65.5)	23 (65.7)

Marital status			
No. (%)			
Single/divorced/widow	20 (52.6)	13 (44.8)	10 (28.6)
Cohabiting/married	18 (47.4)	16 (55.2)	25 (7.4)
Missing	5		

**Table 2:** Means and standard deviations (S.D.s) on the BDI, BAI, and PDS

Questionnaire	PTSD pre-treatment group (*N* = 43)	Anxious control group (*N* = 29)	Non-patient control group (*N* = 31)
BDI	22.23 (9.76)^a^	19.86 (10.67)^a^	4.80 (4.96)^b^
BAI	25.56 (12.99)^a^	29.17 (12.83)^a^	4.80 (4.30)^b^
PDS	32.06 (8.08)^a^	–	3.23 (5.36)^b^

*Note*: Means with different superscripts differ significantly (*p* < 0.05), only 16 participants in the non-patient control group completed the PDS, therefore for this measure *N* = 16.

**Table 3:** Means and standard deviations (S.D.s) on the Probability and Cost Questionnaire

Sub-scale	PTSD pre-treatment (*N* = 43)	Anxious control (probability, *N* = 29) (cost, *N* = 24)	Non-patient control (*N* = 35)
Experiencing a traumatic event			
Probability	33.08 (23.63)^a^	24.05 (19.73)^ab^	16.67 (12.35)^b^
Cost	78.82 (21.16)^a^	77.83 (18.52)^ab^	68.27 (15.40)^b^

Negative world events			
Probability	39.55 (24.72)^a^	30.62 (23.78)^a^	39.43 (19.77)^a^
Cost	37.94 (24.99)^a^	37.86 (26.75)^a^	28.25 (20.16)^a^

Negative life events			
Probability	37.94 (26.34)^a^	31.28 (26.14)^a^	24.69 (15.37)^a^
Cost	71.36 (22.51)^a^	67.19 (17.55)^a^	60.79 (14.22)^a^

Daily hassles			
Probability	41.38 (23.31)^a^	39.61 (24.20)^a^	44.78 (19.95)^a^
Cost	22.33 (15.97)^a^	14.23 (14.75)^b^	14.76 (10.55)^b^

*Note*: Means with different superscripts differ significantly (*p* < 0.05).

**Table 4:** PTSD group pre and post-treatment scores on the Probability and Cost Questionnaire, the PDS, the BAI, and BDI

Measure	*N* = 33	Pre-treatment (mean (S.D.))	Post-treatment (mean (S.D.))	*t* Observed
PDS		31.68 (8.20)^a^	10.96 (11.56)^b^	11.36
BAI		23.42 (12.11)^a^	10.15 (12.43)^b^	6.30
BDI		21.27 (9.59)^a^	12.21 (11.55)^b^	5.64

Probability and cost subscales				
Accidental traumas	Probability	34.05 (22.87)^a^	15.55 (18.97)^b^	6.52
	Cost	80.76 (16.62)^a^	67.79 (23.45)^b^	3.35

Interpersonal violence traumas	Probability	34.43 (27.88)^a^	16.89 (18.46)^b^	4.51
	Cost	83.13 (22.22)^a^	69.75 (25.53)^b^	3.00
				

Negative life events	Probability	39.43 (26.86)^a^	20.77 (25.63)^b^	4.95
	Cost	74.29 (21.93)^a^	60.22 (22.40)^b^	3.78
				

Negative world events	Probability	42.36 (25.67)^a^	29.40 (24.52)^b^	4.21
	Cost	39.52 (25.25)^a^	33.38 (24.88)^b^	1.82

Daily hassles	Probability	41.96 (23.55)^a^	35.86 (25.60)^b^	1.79
	Cost	23.24 (15.90)^a^	14.87 (12.79)^b^	3.29

*Note*: Means with different superscripts differ significantly (*p* < 0.05).
